# Lack of Awareness of Sepsis Among Sepsis Survivors in Acute Care

**DOI:** 10.1093/geroni/igaf122.3616

**Published:** 2025-12-31

**Authors:** Katherine Pitcher, Kathryn Bowles, Michael Stawnychy

**Affiliations:** University of Pennsylvania School of Nursing, Philadelphia, Pennsylvania, United States; University of Pennsylvania School of Nursing, Philadelphia, Pennsylvania, United States; University of Pennsylvania School of Nursing, Philadelphia, Pennsylvania, United States

## Abstract

Sepsis is a life-threatening disease with high 30-day hospital readmission rates (17% to 35%). Sepsis disproportionately affects older adults (≥60 years), who often experience new functional limitations and cognitive impairment after sepsis. Timely surveillance post-discharge has been shown to reduce readmissions in this population. Prevailing nursing theories—Meleis’s Transitions Theory and Riegel’s Self-Care Theory—emphasize the importance of patient awareness and reflection to support effective transitions and self-care. However, patients may not be aware of the sepsis diagnosis. Sepsis survivors who were offered home health care (HHC) were surveyed for a study examining illness representation and decision-making around HHC and outpatient follow-up. Quantitative data collection was guided by theories of illness representation, dyadic illness management, and social determinants of health. Among 179 sepsis survivors (mean age 61.8±15.6 years, 48.6% female, 55.9% White), 41.3% of sepsis survivors were unaware that they had been diagnosed with sepsis. Sepsis survivors who were unaware of their diagnosis were more likely to be male and Black, and more likely to have required intensive care, than those who were aware of their diagnosis. Unaware patients also received timely HHC at lower rates and were readmitted within 30 days at higher rates than those who knew their diagnosis. These findings reveal sepsis survivors’ lack of awareness of their diagnosis as a critical barrier to post-discharge engagement and challenge assumptions embedded in transitional care models. A high rate of poor diagnostic communication highlights an urgent need for enhanced sepsis communication strategies and targeted education for patients.

